# Indicators used in livestock to assess unconsciousness after stunning: a review

**DOI:** 10.1017/S1751731114002596

**Published:** 2014-10-30

**Authors:** M. T. W. Verhoeven, M. A. Gerritzen, L. J. Hellebrekers, B. Kemp

**Affiliations:** 1 Wageningen University and Research Centre, Livestock Research, PO Box 65, 8200 AB Lelystad, The Netherlands; 2 Adaptation Physiology Group, Department of Animal Sciences, Wageningen University, PO Box 338, 6700 AH Wageningen, The Netherlands; 3 Faculty of Veterinary Medicine, Utrecht University, PO Box 80154, 3508 TD Utrecht, The Netherlands

**Keywords:** animal welfare, livestock, slaughter, stunning, unconsciousness

## Abstract

Assessing unconsciousness is important to safeguard animal welfare shortly after stunning at the slaughter plant. Indicators that can be visually evaluated are most often used when assessing unconsciousness, as they can be easily applied in slaughter plants. These indicators include reflexes originating from the brain stem (e.g. eye reflexes) or from the spinal cord (e.g. pedal reflex) and behavioural indicators such as loss of posture, vocalisations and rhythmic breathing. When physically stunning an animal, for example, captive bolt, most important indicators looked at are posture, righting reflex, rhythmic breathing and the corneal or palpebral reflex that should all be absent if the animal is unconscious. Spinal reflexes are difficult as a measure of unconsciousness with this type of stunning, as they may occur more vigorous. For stunning methods that do not physically destroy the brain, for example, electrical and gas stunning, most important indicators looked at are posture, righting reflex, natural blinking response, rhythmic breathing, vocalisations and focused eye movement that should all be absent if the animal is unconscious. Brain stem reflexes such as the cornea reflex are difficult as measures of unconsciousness in electrically stunned animals, as they may reflect residual brain stem activity and not necessarily consciousness. Under commercial conditions, none of the indicators mentioned above should be used as a single indicator to determine unconsciousness after stunning. Multiple indicators should be used to determine unconsciousness and sufficient time should be left for the animal to die following exsanguination before starting invasive dressing procedures such as scalding or skinning. The recording and subsequent assessment of brain activity, as presented in an electroencephalogram (EEG), is considered the most objective way to assess unconsciousness compared with reflexes and behavioural indicators, but is only applied in experimental set-ups. Studies performed in an experimental set-up have often looked at either the EEG or reflexes and behavioural indicators and there is a scarcity of studies that correlate these different readout parameters. It is recommended to study these correlations in more detail to investigate the validity of reflexes and behavioural indicators and to accurately determine the point in time at which the animal loses consciousness.

## Implications

This review evaluates the different ways in which unconsciousness after stunning is assessed and weighs the pros and cons of these methods. Assessing unconsciousness is performed in a variety of ways, depending on species as well as the method of stunning. Assessing brain activity by way of electroencephalogram (EEG) analysis is suggested to be the most objective method to evaluate unconsciousness, but this is only applied in experimental set-ups. Studies in which correlations between the EEG and other indicators are looked at in more detail could provide additional information on the exact time points at which animals lose consciousness after stunning.

## Introduction

European legislation provides laws, rules and procedures regarding the slaughter of livestock (GWvD, 1992; Council Directive 93/119/EC, 1993; Council Regulation (EC) No 1099/2009, 2009). Article 4 of Council Regulation (EC) No 1099/2009 describes the mandatory pre-slaughter stunning, with exception of particular methods of slaughter prescribed by religious rites, to ensure unconsciousness and insensibility to prevent unnecessary suffering of animals. There is no consensus about the extent to which slaughter of conscious, meaning sensible and/or aware, animals causes them pain and distress. It is claimed that when a clean incision is made with an exquisitely sharp knife, significant pain and distress are avoided (e.g. Grandin, 1994; Rosen, 2004). Johnson *et al*. (2012) suggest that massive stimulation of all sensory nerves after the neck cut may lead to shock and distress that would be experienced as pain for the duration of consciousness. Until now, neurophysiological methodology has not provided the ultimate answer to this issue. Because animals are considered not to experience pain when unconscious, it is important to validly determine unconsciousness after stunning. Stunning methods most frequently applied include mechanical stunning (captive bolt), applying an electrical current through the head of the animal or by immersion in a mixture of gasses consisting of (low level) oxygen (O_2_), carbon dioxide (CO_2_), argon (Ar) and/or nitrogen (N_2_). For all stunning methods, it is critical to determine the onset and duration of unconsciousness. Available data from different livestock species to examine the different methods used to assess unconsciousness, include reflexes and behavioural indicators. Less used in practice, but considered the most objective method for the assessment of unconsciousness, involves the evaluation of brain activity as presented in an electroencephalogram (EEG). The possibilities and limitations of the use of EEG for this purpose are further elaborated upon in this manuscript.

## Consciousness and unconsciousness

Consciousness is defined in many different ways, but in general is associated with the awake state and the ability to perceive, interact and communicate with the environment and others (Zeman, 2001). The opposite state, that is, unconsciousness, is defined as: ‘a state of unawareness (loss of consciousness) in which there is temporary or permanent disruption to brain function. As a consequence of this disruption, the unconscious animal is unable to respond to normal stimuli, including pain’ (EFSA, 2006). Disruption of brain function can occur as a result of brain concussion, administration of anaesthetics, anoxia or an electroconvulsive shock (Lopes da Silva, 1982). Some authors prefer the term insensibility over unconsciousness, as they find it less anthropomorphic (Blackmore and Delany, 1988). Insensibility refers to the complete inability to experience any sensations, including unpleasant sensations such as pain (Hemsworth *et al.*, 2009). Pain is defined as ‘an unpleasant sensory and/or emotional experience associated with actual or potential tissue damage, or described in terms of such damage’ (Merskey, 1986). Pain is considered a conscious experience and needs to be avoided during the slaughter process. The term unconsciousness, as used in this review, also includes insensibility. Stunning of animals aims at inducing unconsciousness and thus insensibility, which lasts until the animal is dead. An animal is considered dead when: ‘respiration and blood circulation have ceased as the respiratory and circulatory centres in the medulla oblongata are irreversibly inactive. Because of the permanent absence of nutrients and O_2_ in the brain, consciousness is irreversibly lost’ (EFSA, 2004). During the slaughter process, regular checks should be carried out to ensure that the animal does not present any signs of consciousness or sensibility in the period between the end of the stunning process and death (Council Regulation (EC) No 1099/2009, 2009).

Brain regions that are involved in consciousness are the cerebral cortex and thalamus, together forming the thalamocortical complex, which is regulated by the brainstem. A well-functioning brainstem and thalamus are essential for the maintenance of consciousness and damage to (one of) these regions can cause rapid loss of consciousness (Gregory and Shaw, 2000). However, localised lesions in the cortex, for instance in the sensory cortex, do not necessarily cause unconsciousness, but may only change specific features such as colour vision or the way visual objects and faces are interpreted (Seth *et al.*, 2005). The central core of the brainstem is formed by the reticular formation, a large network of neural tissue located in the central region of the brain stem. The reticular formation receives sensory information from the cortex and several subcortical regions and its axons project to the cerebral cortex, thalamus and spinal cord. The reticular formation plays not only a role in sleep and arousal, but also in attention, muscle tone, movement and various vital reflexes (Carlson, 2007). When the reticular formation fails, the cerebral cortex will be switched off or cannot be switched on. When the cortex is (functionally) damaged, neuronal integration of signals from the central nervous system necessary for conscious perception and subjective experience cannot occur. The disruption of normal electrical brain activity is considered to be incompatible with consciousness (Savenije *et al.*, 2002; Lambooij, 2004; Adams and Sheridan, 2008). To maintain consciousness, a constant supply of O_2_ and energy to the brain and continuous removal of metabolic waste, such as CO_2_, is needed. If one of the mechanisms fails, for instance due to stunning, an animal will become unconscious (Adams and Sheridan, 2008).

## Time to and duration of unconsciousness

In a large-scale study by von Wenzlawowicz *et al*. (2012), stunning effectiveness was assessed in over 37 000 pigs and cattle, stunned by different methods. The mean percentages for animals showing signs compatible with insufficient stunning ranged from 3% to 14%, depending on the stunning method and with a high variability between slaughter plants. Gregory (2008) found that 8% of electrically stunned cattle (*n*=67) were not deeply stunned and showed signs of consciousness at 20 and 90 s post stunning. If stunning is reversible, the chance for recovery should be minimised and the stun-to-stick interval should be kept to a minimum to prevent recovery during exsanguination. With electrical stunning in pigs, an interval under 15 s was recommended, where after exposure to gas a stun-to-stick interval of 25 to 45 s was advised, depending on the gas mixture and concentration used (Anil, 1991; Raj, 1999). Recommendations on the duration of stun-to-stick interval depend on different factors including the amount of current or concentration of gas used and the exposure time. When the stun is found not to be effective, the animal should be re-stunned as soon as possible. Animals that are conscious at time of the neck cut lose consciousness as a consequence of the severe decrease in cerebral blood flow leading to a rapid onset of disorganised brain function and thus unconsciousness (Mellor *et al.*, 2009). Sheep and poultry lose spontaneous brain activity after on average 14 and 23 s when both carotid arteries are severed (Gregory and Wotton, 1984 and 1986). In cattle, however, consciousness after the neck cut is prolonged, as the vertebral arteries, which are not severed by the neck cut, supply blood to the circle of Willis and play a direct role in the blood supply to the brain (Baldwin and Bell, 1963). Cattle lose spontaneous brain activity 75±48 s post neck cut (range 19 to 113 s), but Newhook and Blackmore (1982) suggested possible intermittent sensibility for up to 123 to 323 s after slaughter in cattle (Daly *et al*., 1988). The time to loss of consciousness in non-stunned animals, emphasises the need to verify unconsciousness after stunning and take sufficient time for full bleed out before the start of carcass processing, especially in cattle.

## Assessing unconsciousness

Unconsciousness, caused by temporary or permanent disruption to the brain, is generally assessed by the observation of behavioural indicators, which are internally coordinated responses to internal or external stimuli (Levitis *et al.*, 2009). They include reflexes originating from the brain stem (e.g. eye reflexes) or spinal cord (e.g. pedal reflex) and behavioural indicators such as loss of posture, vocalisation and rhythmic breathing. In an experimental set-up, the assessment of brain activity as presented in an EEG, derivatives of the EEG, and evoked potentials can be used to assess unconsciousness.

### Reflexes

Reflexes are automatic, stereotyped movements that are produced as the direct result of a stimulus and are mediated by the central nervous system (Carlson, 2007). The presence of central reflexes are indicators of consciousness that are linked to functioning of the brain stem or spinal cord. Brain stem reflexes are regulated by 12 pairs of cranial nerves that enter and exit the brain and are not under cortical control. Two cranial nerves (I and II) enter from the forebrain and the other nerves (III to XII) enter and exit from the brain stem (Carlson, 2007; Rubin and Safdieh, 2007). Brain stem reflexes that are used to assess unconsciousness after stunning in livestock are cornea or blinking, palpebral, pupillary light and threat reflex. The cornea reflex causes involuntary blinking of the eyelids in response to stimulation of the cornea and is in general the last reflex to be lost in anaesthetised animals (Dugdale, 2010). The palpebral reflex also results in blinking as a response to touching the medial canthus of the eye and disappears earlier than the cornea reflex in anaesthetised animals. Both the cornea and palpebral reflex require a functional afferent cranial nerve V (trigeminal) and efferent cranial nerve VII (facial) and the relevant eye muscles to function adequately (Adams and Sheridan, 2008). The pupillary light reflex is tested by letting light fall on the eye and observing whether the pupil adapts to it. The reflex is controlled by cranial nerves II (optic) and III (oculomotor) and is not considered a reliable reflex during exsanguination, as exsanguination interferes with the blood supply to the retina (Blackman *et al.*, 1986). When testing the threat reflex, an object (finger or pencil) suddenly approaches the eye and a conscious animal will close its eye or withdraw the head. This reflex requires a functional efferent cranial nerve VII (facial) and integration of the motor cortex, but is not often applied, as it requires the eye to be open. Focused eye movement, not a reflex, is considered a definite sign of consciousness, as it needs cortical activity for perception and controlled motor activity from the eyeball muscles (Grillner *et al.*, 2008; Vogel *et al.*, 2011). It is pointed out that positive eye reflexes alone do not necessarily indicate consciousness, as positive brain stem reflexes might occur on the basis of residual brain stem activity and do not distinguish clearly between consciousness and unconsciousness (Anil, 1991). This especially holds true for animals that are electrically stunned, which was documented as early as 80 years ago (Roos and Koopmans, 1936; Blackmore and Delany, 1988; von Holleben *et al.*, 2010). In both sheep and calves, brain stem reflexes were present long after electrical stunning, even though the EEG was suppressed or iso-electric (Anil, 1991; Anil and McKinstry, 1991). On the other hand, eye reflexes may be inhibited after electrical stunning, whereas the cerebral cortex still functions and the animal may be conscious (Blackmore and Delany, 1988). There is no literature available on the frequency of such incidences and its risk for animal welfare is therefore difficult to estimate. After effective captive bolt stunning, however, no eye reflexes should be present, because of the brain trauma produced (Finnie, 1995; Gregory and Shaw, 2000). Thus, cranial nerve reflexes can be good indicators for impaired midbrain or brain stem activity, but only work reliably in one way: when absent, it is very likely that the animal is unconscious, but when they are present, the animal is not necessarily conscious. Spinal reflexes include stretch and flexor reflexes. The stretch reflex, a monosynaptic reflex, is the most basic reflex and plays an important role in control of posture. It does not involve the brain and is therefore not used to assess unconsciousness (Carlson, 2007). The flexor reflex, a polysynaptic reflex, involves activation of nociceptors and is used to assess unconsciousness (Anil, 1991; Erasmus *et al*., 2010). An example of a flexor reflex is the pain withdrawal reflex, which is elicited by applying a painful stimulus to the animal, such as a nose or ear prick. In a survey on expert opinion, the pain withdrawal reflex was ranked high, and thus valued highly, as an indicator to assess unconsciousness after all types of stunning (Gerritzen and Hindle, 2009). The pedal reflex is elicited by, for instance, pinching the skin between the toes of an animal. This reflex is often used for assessment of depth of anaesthesia in laboratory animals, such as rodents and rabbits, but is only occasionally applied in livestock after stunning, as all spinal reflexes are difficult to assess when animals exhibit convulsions or body movements (Tidswell *et al.*, 1987). This especially holds true for animals that are physically stunned, for example, captive bolt stunning, when there is lack of inhibition from the brain and spinal reflexes may occur more vigorously (Blackmore and Delany, 1988). Again, electrically stunned animals may exhibit this reflex long after losing consciousness and the reflex may occur more vigorously when the animal is handled (Blackmore and Newhook, 1982). The righting reflex refers to any reflex that tends to bring the body into its normal upright position. It is often assessed when animals are removed from the stunning box or are hung to the bleeding rail and is also referred to the head righting reflex. This reflex is also difficult to assess when animals exhibit convulsions or involuntary body movements (Blackmore and Newhook, 1982; Anil, 1991). Table 1 shows an overview of the different brain stem and spinal reflexes used to assess unconsciousness after stunning.

**Table 1:** Reflexes used to assess unconsciousness in livestock after stunning

		Present in animals that are^1^			
Reflex	Definition	Conscious	Unconscious	Based on	Remarks
Brain stem reflexes	Reflexes that originate from the brain stem			Functional cranial nerves originating from the brain stem	Reflexes may be present in animals that are unconscious, depending on the method of stunning (Gerritzen and Hindle, 2009) Absence of these reflexes though are considered valuable indicators for assessing unconsciousness (von Holleben *et al.*, 2010) Cannot be tested when seizures occur (Blackmore and Delany, 1988)
Cornea reflex	Involuntary blinking in response to stimulation of the cornea	+ (−)	− (+)	Functional cranial nerves V and VII and eye muscles	One of the most commonly used reflexes after stunning In general the last reflex to be lost in anaesthetised animals (Dugdale, 2010) May be present after electrical stunning, but never after effective captive bolt stunning (Roos and Koopmans, 1936; Gregory and Shaw, 2000)
Palpebral reflex	Involuntary blinking in response to touching the medial canthus of the eye	+ (−)	− (+)	Functional cranial nerves II and III and eye muscles	Disappears earlier than the cornea reflex in anaesthetised animals (Dugdale, 2010)
Pupillary light reflex	Narrowing of the pupil in response to light that falls on the retina	+	−	Functional cranial nerves V and VII and eye muscles	Considered of little value during exsanguination, as the blood supply to the retina is restricted during this period (Blackman *et al*., 1986) Pupillary dilatation is considered a sign of total brain dysfunction (Blackman *et al.,* 1986) May be absent in paralysed, though conscious animals (Blackmore and Delany, 1988)
Threat reflex	Involuntary blinking or withdrawal of the head in response to bringing a finger or hand with speed towards the eye of an animal	+	−	Functional cranial nerve VII, eye muscles and integration with motor cortex	Cannot be tested when the eyes are closed
Spinal reflexes	Reflexes that originate from the spinal cord			Require a functional spinal cord, but do not necessarily require cerebral coordination	May occur more vigorously when there is lack of inhibition from the brain (e.g. captive bolt stunning; Blackmore and Delany, 1988)
Pain withdrawal reflex	Withdrawal of the body part that has had a painful stimulus applied to	+ (−)	− (+)		In a survey on expert opinion, the pain withdrawal reflex was ranked high, and thus valued highly, as an indicator to assess unconsciousness after all types of stunning (Gerritzen and Hindle, 2009)
Pedal reflex	Withdrawal of the foot in response to pinching (the skin between) the toes of an animal	+ (−)	− (+)		Difficult to assess when convulsions occur Not easy to perform in all species. Mainly used in poultry
Righting reflex	Bringing the body into its normal position when taken out of its normal upright position	+ (−)	− (+)		Difficult to assess when convulsions occur (Blackmore and Newhook, 1982; Anil, 1991)

1 Presence and absence of reflexes are presented as follows:+=present, −=absent, (+)=may be present, (−)=may be absent.

### Behavioural indicators

Loss of posture, the inability of the animal to remain in an initial standing or sitting position, is considered a valuable indicator as it is often the first sign to be lost after successful stunning and indicates that the cerebral cortex is no longer able to control posture (Raj *et al.*, 1992; Raj and Gregory, 1996; Llonch *et al.*, 2013). Both mechanical and electrical stunning should lead to immediate collapse (AVMA, 2013). Nystagmus, involuntary rapid horizontal eye flickering, is caused by damage to the vestibular, labyrinthine or central nervous system and was more present in cattle that had a shallow depth of concussion following captive bolt. It was observed in only 3% of 1608 cattle, but was associated with a greater chance of rhythmic breathing. Its presence could add strength to the conclusion that the depth of concussion has been shallow (Gregory *et al*., 2007). In a study by Bourquet *et al*. (2011), nystagmus was observed in one out of 95 captive bolt shot cattle. This animal was reshot, and this supported the study by Gregory *et al.* (2007), which indicated that when nystagmus was observed, there was a one in three chance that the quality of the stun was insufficient. Nystagmus may occur as a result of electrical stunning (Grandin, 2002), but in CO_2_-stunned pigs, nystagmus was not observed once (Atkinson *et al*., 2012). It is stated that under no circumstances should a stunned animal vocalise, as vocalisation after stunning indicates consciousness and probably distress and pain (Grandin and Smith, 2004; Gouveia *et al.*, 2009). A large network of brain regions is involved in the production of vocalisations, including the frontal lobe and primary motor cortex and vocalisations are considered a conscious response (Carlson, 2007). The involuntary passage of air along the vocal cords, however, may cause sounds that can be mistaken for vocalisations. Absence of vocalisations on the other hand, is certainly no guarantee for absence of pain or distress, as the occurrence of vocalisations also depends on the species. A sheep often does not vocalise when injured, where a pig will scream loudly (Broom, 2001; EFSA, 2004). Grandin (2002) believes an animal to be unconscious when it shows a limp head and protruding tongue. The tongue is controlled by nerve XII (hypoglossal) and when relaxed this may indicate loss of cranial nerve function. A study by Gregory *et al*. (2007) showed that a protruding tongue was not associated with depth of concussion after captive bolt stunning, but was proposed as indicator following exsanguination, when 40% of the cattle had a protruding tongue while hanging on the bleeding rail. Similarly, relaxation of the jaw may be taken into account, but can be observed in conscious animals (Gregory *et al.*, 2009). Both jaw relaxation and tongue protruding are not used as single indicators to assess unconsciousness, but can support other indicators of unconsciousness (Grandin, 2002; von Holleben *et al.*, 2010). Beside the important role regarding consciousness, the brain stem also houses the regulatory centres for respiratory and circulatory systems. Rhythmic breathing movements after stunning indicate that the corticospinal, ventral and lateral columns of the spinal cord are still intact and may thus indicate consciousness (Mitchell and Berger, 1975). The presence of rhythmic breathing after stunning is generally accepted to indicate that an animal may not be fully unconscious and is thought to be one of the first signs of recovery after CO_2_ and electrical stunning (Gerritzen and Hindle, 2009; Anastasov and Wotton, 2012). In captive bolt stunned cattle, rhythmic breathing immediately disappears after an effective shot because of axonal injuries to the brainstem (Finnie *et al.*, 2000). The occurrence of convulsions, observed as uncontrolled movements of the body, indicates effective stunning in electrical or mechanical stunned animals, but also occur in unconscious animals that are gas stunned (Adams and Sheridan, 2008; Marzin *et al.*, 2008; von Holleben *et al.*, 2010). These convulsions are thought to be incompatible with consciousness due to the absence of higher motor control (Lambooij, 2004). They can, however, sometimes be mistaken for rhythmic breathing, as they can occur as almost rhythmic body movements (Wotton and Sparrey, 2002). Gagging refers to low-frequency inhalations with the neck positioned towards the front legs and occasional emission of sounds similar to snoring and is considered an indicator of deep unconsciousness (Rodríguez *et al.*, 2008). Gasping is seen when an animal takes deep breaths through an open mouth and is considered an indicator of onset of breathlessness during CO_2_ stunning, which continues long after loss of consciousness even when brain activity is no longer recorded, but may also occur after electrical stunning (Blackmore and Petersen, 1981; Newhook and Blackmore, 1982; Grandin, 2013). Interpretation of all individual indicators mentioned above can be doubtful unless supported by other information (Blackmore, 1984; Gerritzen and Hindle, 2009; Anastasov and Wotton, 2012). Table 2 shows an overview of the different behavioural indicators used to assess unconsciousness after stunning.

**Table 2:** Behavioural indicators used to assess unconsciousness in livestock after stunning

		Present in animals that are^1^			
Indicator	Definition	Conscious	Unconscious	Based on	Remarks
Loss of posture	The inability of the animal to remain in an initial standing or sitting position	− (+)	+ (−)	Absent when cerebral cortex can no longer control posture	Both mechanical and electrical stunning should lead to immediate collapse (AVMA, 2013) More pronounced in gas stunning and starts before loss of consciousness (Raj *et al.*, 1992; Raj and Gregory, 1996; Llonch *et al.*, 2013)
Nystagmus	Involuntary rapid horizontal eye flickering	− (+)	− (+)	Damage to the vestibular, labyrinthine or central nervous system	Should be absent, but its presence after captive bolt stunning could add strength to the conclusion that the depth of concussion has been shallow (Gregory *et al*., 2007) May occur as a result of electrical stunning (Grandin, 2002) Not observed after CO_2_ stunning in pigs (Atkinson *et al*., 2012)
Vocalisations	Voluntary sounds made by the vibration of vocal folds modified by the resonance of the vocal tract	+ (−)	−	A large network of brain regions that is involved in the production of vocalisations	Can be observed while assessing other indicators and should not be present after stunning (Grandin and Smith, 2004; Gouveia *et al.*, 2009) Occurrence of vocalisations is very dependent of the species. (Broom, 2001; EFSA, 2004)
Focused eye movement	Accommodation of the eye	+	−	Functional brain stem and cortex	Not used as an indicator itself, though may be observed when assessing other indicators Considered a definite sign of consciousness (Vogel *et al.*, 2011)
Protruding tongue	Tongue hanging from the mouth	−	+ (−)	Absence of functional cranial nerve XII and loss of control of tongue muscles	Little reported in literature. Based on expert opinion mainly and may be gender dependent in captive bolt stunned in cattle (Grandin, 2002; EFSA, 2004; Gregory *et al*., 2007)
Relaxed jaw	No tension on the jaw	−	+ (−)	Absence of functional cranial nerve V and control of jaw muscles	Little reported in literature. Based on expert opinion mainly and may depend on stunning method (Grandin, 2002; EFSA, 2004; Gregory *et al*., 2009)
Limp head/no neck tension		(−)	(+)	Absence of functional cranial nerve XI and control of neck muscles	May be masked when neck muscles are severed
Rhythmic breathing	Breathing consisting of rhythmic in and exhalation	+	−	Intact corticospinal, ventral and lateral columns of the spinal cord	Considered the first sign of potential return of consciousness following stunning (Gerritzen and Hindle, 2009; Anastasov and Wotton, 2012) Difficult to assess when convulsions occur
Convulsions	Uncontrolled involuntary contraction of muscles. Clonic (uncontrolled jerking) and tonic (rigid) activity	−	+	Absence of higher centre motor control	A lot of variation is seen between animals (Anil, 1991) Not thought to be compatible with consciousness (Lambooij, 2004) Can be misinterpreted as rhythmic breathing (Wotton and Sparrey, 2002)
Gagging	Low-frequency inhalations with the neck towards the front legs and occasional emitting of sounds similar to snoring	−	+	Functional cranial nerves IX and X and control of pharynx muscles	Considered an indicator of deep state of unconsciousness (Rodríguez *et al.*, 2008)
Gasping	Deep breaths taken non-rhythmically through an open mouth	+	+	Suppression of neuronal activities aimed at respiration in the pons and the occurrence of certain mechanisms in the medulla	Is a first indicator of onset of breathlessness and may persist even when no brain activity is recorded anymore (Blackmore and Petersen, 1981; Newhook and Blackmore, 1982) May be present after electrical or CO_2_ stunning (Grandin, 2013)

1 Presence and absence of reflexes are presented as follows:+=present; −=absent; (+)=may be present; (−)=may be absent.

### Brain activity (EEG)

When monitoring brain functioning, activity can be presented in an EEG, which displays electrical activity derived from electrodes attached to various locations on the surface of the head. The EEG is considered the most objective method for assessing unconsciousness and reflects the sum of underlying electrical activity of populations of neurones supported by glia cells (Murrell and Johnson, 2006). There are four different types of wave patterns in the EEG that can be distinguished based on their respective frequencies and that are related to the state of consciousness: *δ* (0 to 4 Hz), *θ* (4 to 8 Hz), *α* (8 to 12 Hz) and *β* (>12 Hz) waves. Both *δ* and *θ* (slow wave) activity is related to sleep or reduced consciousness. *α* activity is prominent in subjects that are conscious, but mentally inactive (closing eyes and relaxation) and *β* waves are associated with active movements and increased alertness (Kooi *et al.*, 1978; Niedermeyer *et al*., 2011). Depending on the method of stunning, the EEG shows a characteristic pattern of change when animals lose consciousness. Generally, an increase in low frequency activity is accompanied by an increase in amplitude. When neurons depolarise at the same time or frequency, they fire in a synchronised fashion creating slow high amplitude waves as seen in unconscious states suggesting a depression of the reticular formation (Lopes da Silva, 1982). Consciousness on the other hand is characterised by high frequency (*α* and *β*), low amplitude waves (Seth *et al.*, 2005). When looking more specifically at EEG wave patterns, the EEG can be broken down in different time segments, better known as epochs. These epochs can be analysed for frequency (Hz), amplitude (µV) and power (µV^2^), together representing the amount of activity in the brain. Four stages of EEG can be distinguished during the process of stunning and slaughter and are related to the level of consciousness, namely: active, transitional, unconscious and iso-electric (flat) EEG. In the first (active) stage, normal awake activity is recorded with high frequency, low amplitude waves, indicating the animal is conscious. In the second (transitional) stage, the amplitude of the EEG increases together with a decrease in frequency. When these changes become more profound, the animal is considered unconscious. When loss of consciousness progresses, the EEG turns iso-electric and brain activity is no longer recorded (Gibson *et al.*, 2007; McKeegan *et al.*, 2007). The exact moment when unconsciousness sets in, based on the EEG, is difficult to determine as changes are often gradual. The iso-electric EEG, however, is never compatible with consciousness. There is no consistency in the literature regarding the number of stages used in the assessment of unconsciousness. Other research may only differentiate between the stages conscious and unconscious or contrary, use additional stages besides the four mentioned above.

### Derivatives of the EEG

Another way of analysing raw EEG data, next to visual appraisal of the EEG, would be to compute a Fast Fourier Transformation (FFT). The output thereof represents the frequency composition of the signal, or alternatively formulated, how much power is presented in the different frequency bands. The principle is similar to defining the EEG in different EEG types that consist of slow or fast waves with high or low amplitudes (Davidson, 2006). Further (automatic) calculations of the FFT can lead to EEG derivatives presenting a single value or percentage that is easier to standardise.

Derivatives of the EEG include: the total power (*P*_tot_), which is the area underneath the frequency spectrum curve, the medium frequency (*F*_50_), which is the frequency below which 50% of the total power is located and the spectral edge frequency (*F*_95_), which is the frequency below which 95% of the power is located. These readout parameters are considered quantitative tools to describe changes in EEG activity (Murrell and Johnson, 2006). An initial increase in *P*_tot_ may represent a loss of functional cerebrocortical activity when amplitudes of EEG waves increase because of synchronised firing of neurons. But as the level of unconsciousness deepens, a decrease in all three derivatives is seen (Bager *et al.*, 1992; Martoft *et al.*, 2001). In electrically stunned livestock, an increase in power of all frequency bands is first observed in the first 5 to 15 s post-stun because of initial epileptiform activity (Velarde *et al.*, 2002; Beyssen *et al.*, 2004). Automatic FFT is applied during human surgeries and on a smaller scale during animal surgeries, where the raw EEG and its FFT are used to assess anaesthetic depth. Established anaesthesia monitors are used to assess depth of anaesthesia, but they differ in the algorithm used to analyse the EEG (Bruhn *et al.*, 2006). To the authors’ knowledge, only one of such monitors, namely the Index of Consciousness or IoC, has been used in a study concerning stunning in animals. During gas stunning of pigs, the raw EEG was recorded and based on that data a dimensionless variable (IoC) was calculated (Llonch *et al.*, 2013). This variable ranges from 100 (awake) to 0 (iso-electric) and decreases with increasing anaesthetic and sedative depth. Values between 40 and 60 are suggested to represent an adequate hypnotic effect of the subject under general anaesthesia (Grover and Bharti, 2008). In the study by Llonch *et al*. (2013), time to loss of posture occurred almost 20 s earlier then the accompanying decrease in IoC. A delay in IoC reading, compared with loss of balance, was also seen in pigs anaesthetised with propofol, but with a delay of only 7 s (Llonch *et al.*, 2011). Muscular excitations that occur during CO_2_ stunning probably affected the IoC calculation, as movement artefacts are known to influence EEG data and calculations made in anaesthesia monitors (Teplan, 2002). This is one of the reasons offline calculation is used to more adequately compare and correlate brain activity data with behavioural indicators. Though many studies have looked at behavioural indicators or the EEG separately, only a few have studied correlations between these different read-out parameters for assessing unconsciousness. In a study by Benson *et al*. (2012), loss of posture was correlated to the *α*/*δ* ratio extracted from the EEG, in an effort to find a more objective and alternative method (as opposed to loss of posture) to assess loss of consciousness in broilers. A correlation and no difference was found between time to unconsciousness as observed by the two methods, supporting the use of *α*/*δ* ratio as method to assess unconsciousness. The study shows that such correlations can provide additional, more objective data to support the use of behavioural indicators as a measure of unconsciousness and provide details when certain behaviours may be present or absent in an animal that loses consciousness.

### Evoked responses

The EEG recording is also used to assess unconsciousness by way of generating evoked responses. Evoked responses are responses in the EEG following external stimuli (visual, somatosensory or auditory), generated in specific areas of the cerebral cortex, mid brain and brainstem (Schneider and Sebel, 1997; Grover and Bharti, 2008). Evoked responses are frequently used as additional indicators to assess unconsciousness next to behavioural indicators, and have been applied in sheep, cattle, poultry and pigs. No correlations, however, have been calculated for the presence or absence of evoked potentials and presence or absence of behavioural indicators. Though, similar to EEG derivatives, evoked potentials may in this way provide additional support for the use of certain behavioural indicators. As for now, evoked responses are only used in experimental set-ups. Rapid changes in consciousness are difficult to observe with evoked potentials, as repeated stimulation and averaging of data (EEG) is needed to see these changes (Beyssen *et al.*, 2004). Differences in time to loss of consciousness based on the loss of spontaneous EEG or evoked responses have been observed in multiple studies. In hens stunned with different gas mixtures, evoked responses were observed to disappear ∼15 s after the EEG became suppressed, but almost 30 s before the occurrence of an iso-electric EEG (Raj *et al.*, 1991 and 1992). In poultry slaughtered by nine different methods, all without prior stunning, spontaneous brain activity was lost after 23 to 233 s, where visual evoked potentials were lost after 90 to 349 s (Gregory and Wotton, 1986). The loss of somatosensory evoked potentials was also recorded before an iso-electric EEG, but after a suppressed EEG in gas-stunned turkeys (Raj and Gregory, 1993). The presence of an evoked response implies that the afferent pathways to the higher brain centres are intact, but not necessarily that the animal is aware of the stimulus (Raj *et al*., 1991). Visual evoked potentials have been observed in, for instance, anaesthetised animals (Gregory and Wotton, 1986; Gregory, and Wotton, 1989). Conversely, the absence of evoked potentials may not always guarantee unconsciousness (Anil *et al.*, 2000). Gregory and Wotton (1990) looked at the effects of multiple electrical stunning currents on spontaneous physical activity and evoked responses and found that the loss of somatosensory evoked potentials indicated a deeper level of unconsciousness than absence of neck tension. All these studies show that the use of different methods to assess unconsciousness may lead to different findings regarding the time to loss of consciousness. The use of absence of evoked responses or iso-electric EEG, may provide more conservative times to loss of consciousness compared with loss of spontaneous EEG. The indicators based on brain activity that can be used to asses unconsciousness after stunning are presented in Table 3.

**Table 3:** Indicators based on brain activity as presented in an electroencephalogram (EEG) used to assess unconsciousness in livestock after stunning

		Development in animals that are		
Indicator	Definition	Conscious	Unconscious	Remarks
EEG	Presents electrical activity of the brain	Mainly fast low amplitude (voltage) waves (8 to 30 Hz)	Mainly slow high amplitude (voltage) waves (0 to 8 Hz)	Considered the most objective way of assessing unconsciousness (EFSA, 2004) Takes experience to assess the EEG and at present only possible in experimental set-ups
Derivatives of the EEG	Calculated mathematical readout parameters based on brain activity (EEG)			Easier to standardise than visual analysis of the raw EEG trace
Fast Fourier Transformation	Frequency composition of the signal at a certain time point	More power in the higher frequency bands (8 to 30 Hz)	More power in the lower frequency bands (0 to 8 Hz)	No golden standard in its division, no clear cut-off point (Alkire *et al*., 2008)
Total power (Ptot)	Area underneath the frequency spectrum curve		May be high in an epileptiform insult or when losing consciousness during anaesthesia, but decreases when unconsciousness deepens	No golden standard in its division, no clear cut-off point
Spectral edge frequency (*F*_95_)	Frequency below which 95% of the power is located	Will be higher, because of the power in the higher frequency bands	Will decrease, because of the increasing power in the lower frequency bands	No golden standard in its division, no clear cut-off point
Median frequency (*F*_50_)	Frequency below which 50% of the power is located	Will be higher, because of the power in the higher frequency bands	Will decrease, because of the increasing power in the lower frequency bands	No golden standard in its division, no clear cut-off point
Indexes/anaesthetic depth monitors	Use algorithms to transform raw EEG into a single value representing anaesthetic depth	Increases when the animal regains consciousness	Decreases when the animal loses consciousness	Gives you a single value, easier to interpret Sensitive to artefacts (Teplan, 2002) Based on human data
Evoked responses	Presents electrical activity from the brain in response to external stimuli	Present	Absent	May persist in animals that are unconscious (Gregory and Wotton, 1986). Rapid changes cannot be observed (Beyssen *et al*., 2004)

### Difficulties in the use of EEG

Though the EEG may be considered most objective when assessing unconsciousness, there are some disadvantages to its use. First, there is no golden standard for the way in which the division of stages of consciousness is described and this also limits the use of brain function monitors in differentiating between consciousness and unconsciousness, especially during transitional stages (Alkire *et al.*, 2008). Second, it is difficult to compare EEG values between species and individuals, because of animal variation caused by electrode placement, skull thickness and differences between equipment. Third, the EEG can be influenced by artefacts that are animal related (eye or muscle movements) or technical related (cable movements, impedance fluctuation or 50/60 Hz interference) (Teplan, 2002). Experimental controlled situations provide a significantly better environment to limit these artefact sources than slaughter plants. These artefacts, however, limit possibilities for EEG application as an evaluation method in slaughter plants at this stage.

## Conclusion

This literature review shows that there is a wide range of indicators available to assess unconsciousness in livestock after stunning. In general, pathophysiology of the consequences of the stunning method should be taken into account when assessing unconsciousness, as applicability and reliability of the different indicators vary per stunning method. When physically stunning an animal, for example, captive bolt, most important indicators are posture, righting reflex, rhythmic breathing and the corneal or palpebral reflex that should all be absent when the animal is unconscious. Spinal reflexes are difficult as a measure of unconsciousness with this type of stunning, as they may occur more vigorous. For stunning methods that do not physically destroy the brain, for example, electrical and gas stunning, most important indicators are posture, righting reflex, natural blinking response, rhythmic breathing, vocalisations and focused eye movement that should all be absent when the animal is unconscious. Brain stem reflexes such as the cornea reflex are difficult as measures of unconsciousness in electrically stunned animals, as when present they may reflect residual brain stem activity and not necessarily consciousness. It is highly recommended to use multiple indicators to definitively assess and determine unconsciousness before starting invasive dressing procedures such as scalding or skinning. The EEG is generally considered to be a most reliable indicator for assessing unconsciousness, but is (the most) difficult to apply during slaughtering because of technical- and animal-related artefacts that can occur. Furthermore, the lack of a golden standard for determining (un)consciousness makes the evaluation of the EEG somewhat subjective. It is recommended to put further effort into resolving these difficulties so that the EEG can be more easily used in the assessment of unconsciousness after stunning. A substantial number of controlled studies have used the EEG to assess unconsciousness, but only one focussed on the correlation between an EEG derivative and a behavioural indicator. More research in this area should provide additional information on the absence of behavioural indicators in relation to the EEG and validate the use of certain behavioural indicators. Overall, better validated and applicable indicators are needed to reliably and reproducibly assess unconsciousness. These indicators could potentially also provide additional information on the onset of unconsciousness during the transitional period, as at present this is highly subjective, as it is often based on visual appraisal. Knowledge derived from studies using EEG in combination with other indicators in experimental set-ups could subsequently lead to improvements regarding stunning methods and subsequently animal welfare at the slaughter plant.
